# Advances in Targeting Cancer-Associated Genes by Designed siRNA in Prostate Cancer

**DOI:** 10.3390/cancers12123619

**Published:** 2020-12-03

**Authors:** Amirhossein Bahreyni, Honglin Luo

**Affiliations:** 1 Centre for Heart Lung Innovation, St. Paul’s Hospital, 1081 Burrard St, Vancouver, BC V6Z 1Y6, Canada; amirhossein.bahreyni@hli.ubc.ca; 2 Department of Pathology and Laboratory Medicine, University of British Columbia, Vancouver, BC V6Z 1Y6, Canada

**Keywords:** siRNA, prostate cancer, cancer-associated genes, delivery, nanoparticle

## Abstract

**Simple Summary:**

Despite great advancements in early detection and therapeutic strategies, the 5-year survival rate for patients with metastatic prostate cancer remains low (i.e., ~30%). Targeting prostate cancer-associated genes has emerged as a promising treatment for this devastating disease. This review summarizes recent findings in silencing genes that are involved in prostate cancer pathogenesis. Moreover, novel nanotechnology-based platforms for effective delivery of therapeutic RNAs to prostate cancer cells have been discussed. Information provided in this review will benefit both researchers and clinicians to design and develop novel therapeutic approaches for patients suffering from prostate cancer.

**Abstract:**

Short interfering RNAs (siRNAs) have provided novel insights into the field of cancer treatment in light of their ability to specifically target and silence cancer-associated genes. In recent years, numerous studies focus on determining genes that actively participate in tumor formation, invasion, and metastasis in order to establish new targets for cancer treatment. In spite of great advances in designing various siRNAs with diverse targets, efficient delivery of siRNAs to cancer cells is still the main challenge in siRNA-mediated cancer treatment. Recent advancements in the field of nanotechnology and nanomedicine hold great promise to meet this challenge. This review focuses on recent findings in cancer-associated genes and the application of siRNAs to successfully silence them in prostate cancer, as well as recent progress for effectual delivery of siRNAs to cancer cells.

## 1. Introduction

Prostate cancer (PCa) is the most commonplace malignancy in men along with the second most frequent cause of cancer-related death in males in the Western population, just behind lung cancer [1]. In 2020, it is anticipated that 191,930 American men will be diagnosed with prostate cancer and 33,330 patients will die due to this malignancy [2]. Among more than a million new cases diagnosed with PCa worldwide annually, statistics shows that roughly 30% of patients succumb to this disease [3]. Moreover, in light of higher incidence of this abnormality among the elderly and as a result of enhancement in life expectancy, it is estimated that the number of PCa new cases elevates roughly 80% to more than two million annually by 2040 [4,5], making this malignancy a critical health issue, particularly in developed countries [6]. Despite great advancements in current therapeutic strategies for PCa such as surgery, radiotherapy, and androgen deprivation therapy, the 5-year survival of patients with distant tumors remains low (i.e., ~30%) [7,8]. Therefore, it is exigent to identify novel targets for developing more effective treatments to combat this growing concern.

Recent advancements in genetic research have provided numerous innovative tools for treatment of various abnormalities, from inflammation to diverse types of cancers. In line with this, RNA interference (RNAi) has attracted tremendous attention as a novel therapeutic tool in the clinical setting among the research community merely after it was discovered in the 1990s [9,10]. Short interfering RNAs (siRNAs), also recognized as small RNAi, are double-stranded RNAs with 21–25 nucleotides that are applied to silence target genes in cells [11,12]. siRNAs are made of two single sequences, named sense strand (passenger strand) and antisense strand (guide strand), connected by an active protein complex called the RNA-Induced Silencing Complex (RISC) [13,14]. It has been demonstrated that guide strand acts as the guide for the recognition of complementary mRNAs, and the RISC complex forms through their attachment. As soon as the guide strand binds to the target mRNA, the mRNA is cleaved by argonaute 2 (Ago2), the catalytic subunit of the RISC complex [15,16]. siRNAs have gained importance as effectual drugs against specific genes involved in the pathogenesis of different disorders, including viral infections and cancers [9,17]. The review will discuss the potential value of siRNAs in the treatment of PCa and elaborate novel approaches for siRNA delivery.

## 2. siRNA as a Therapeutic Agent in Various Types of Cancers

There is growing evidence proving that siRNAs can be considered as promising therapeutics for many human pathologies, namely cancer, cardiovascular diseases, and other abnormalities of genetic etiology [18,19]. Cancer is the most prevalent genetic disorder, provoked from the alteration in precise genes within cancer cells. Various types of gene mutations, which can either activate oncogenes or inactivate tumor suppressor genes in cells, could result in initiating cancer or even exacerbating it [20]. Therefore, one of the promising ways to tackle different types of malignancies is gene therapy to modulate the expression pattern of particular genes participating in tumor pathogenesis [21]. Since their discovery, siRNAs have attracted tremendous attention as novel cancer therapeutics. In line with this, it is proven that elongation factor 2 kinase (EF2K) is overexpressed in breast cancer of patients with mutations in *BRCA1* (a human tumor suppressor gene), consequently triggering significant tumor growth and poor survival rate. Using siRNAs to target EF2K significantly declines cell proliferation, migration, and invasion of the cancer cells. Additionally, delivery of EF2K-specific siRNAs into *BRCA1*-mutated breast cancer in an orthotopic xenograft model by silica-coated cobalt-ferrite nanoparticles considerably diminishes tumor growth and metastasis [22]. Furthermore, acetylation of ATP-binding cassette transporter E1 (ABCE1), which is often induced by a Tat interactive protein 60 kDa (Tip60), has been found to be increased in the tissues and cells of lung cancer. Gene silence of Tip60 in lung cancer cells by designed siRNA reduces the acetylation of ABCE1, accompanied by reduced tumor weight and volume [23]. Consistent with these findings, it was shown that knockdown of neuromedin U receptor 2, a bioactive and highly conserved neuropeptide, through siRNA significantly enhances paw withdrawal threshold value in bone cancer pain via inactivation of PKC/ERK and PI3K/AKT signaling pathways in comparison with negative controls [24]. These data suggest that using siRNA to silence genes involved in tumor pathogenesis has potential value for cancer treatment. 

Numerous proteins are upregulated in cancers and have crucial roles in cancer development. The precursor of nerve growth factor is one of the proteins that was recently shown to be overexpressed in pancreatic cancer tissues and cell lines. It was reported that knockdown of this specific growth factor by siRNA diminishes cell proliferation, migration, and invasion, and promotes anoikis of pancreatic cancer cells [25]. Similar to these findings, Li et al. [26] showed that water channel proteins (aquaporins), which are responsible for the transportation of water molecules, can participate in tumor cell proliferation and metastasis in breast cancer. It was found that silence of aquaporin-5 via siRNA decreases invasion and migration of cancer cells and augments the chemosensitivity of MCF-7/ADR cells to adriamycin, indicating the effectiveness of siRNAs in breast cancer treatment [26]. Additionally, Tang et al. [27] studied the impact of siRNA knockdown of CT45A1 (cancer-testis antigen family 45 member A1) in cancer therapy and showed that depletion of CT45A1 results in significant inhibition of cell viability, migration, and invasion of lung cancer cells, accompanied by reduced expression of Bcl-2, survivin, matrix metallopeptidase (MMP)-2 and -9, phospho-ERK1/2, and phospho-cAMP-responsive element binding protein-1, along with an increase in Bax expression [27]. Another study evaluated the effect of gene silence of STAT6 on tumor growth [28]. STAT6 is highly expressed in tumor cells and its level is positively correlated with a high rate of cell proliferation and poor prognosis. It was proven that STAT6-specific siRNA decreases viability of both HT-29 colorectal cancer and ZR-75-1 breast cancer cells through inhibiting cell proliferation and initiating apoptosis [28]. In vivo studies substantiated the efficiency of siRNA in knocking down STAT6 and subsequent inhibition of tumor growth [28]. 

siRNAs in combination with other therapeutic agents including chemotherapeutic drugs have exhibited synergetic effects in the treatment of various cancers. It has been shown that gene silencing of Notch-1, Wnt/β-catenin, and STAT3 alone or in combination significantly enhances the chemosensitivity of both doxorubicin-sensitive and -resistant MCF-7 breast cancer cells to doxorubicin, indicating the potential role of siRNA in the enhancement of the anticancer efficacy of conventional therapies [29]. Furthermore, it was reported that siRNA knockdown of Bag-1, an antiapoptotic protein, sensitizes MCF-7 breast cancer cells to apoptosis initiated by chemotherapeutic drugs such as cisplatin or paclitaxel. This combination therapy results in a significant reduction of pro-survival PI3K/Akt/mTOR and ERK1/2 pathways, an enhancement in stress-activated p38 and SAPK/JNK mitogen-activated protein kinase pathways, and an upregulation of tumor suppressors p21 and Rb [30]. Overall, these results indicate that designing and employing siRNAs against oncogenes either alone or in combination with other conventional therapies like chemotherapy can be a promising procedure to combat different malignancies.

## 3. siRNA-Mediated Cancer-Associated Gene Silencing in Prostate Cancer

As alluded to above, RNAi offers a novel and beneficial strategy in the field of cancer therapy. Despite the late discovery, siRNAs have been developed to silence countless genes responsible for diverse PCa hallmarks, including angiogenesis, invasion, and metastasis. In line with this, knockdown of a dual specificity protein kinase TTK, which is involved in chromosome segregation during mitosis, by designed siRNA, has been reported to be able to reduce proliferation, invasion, and migration of PC3 and DU145 PCa cells and initiate cell death [31]. Once applied to tumor-bearing mice, this developed siRNA prevents tumor cells from division in comparison with the control group, signifying a potential target for PCa treatment [31]. It was also reported that knockdown of Rho-associated protein kinase by siRNA significantly reduces migration and invasion of PC-3 and DU145 PCa cells, which are associated with decreased expression of phospho-LIM kinase 1 and MMP-2 [32]. Protein phosphatase 2A (PP2A) is known as an oncoprotein overexpressed in most human malignancies, especially PCa. Growing evidence suggests that this enzyme could be a potential target for PCa treatment. It was validated that siRNA-mediated gene silencing of PP2A significantly elicits sensitivity of PC-3 cells to docetaxel-induced cell growth inhibition and apoptosis [33]. Poly (ADP-ribose) polymerase 1 (PARP1) is a nuclear protein that is involved in various cellular processes and has been revealed to have prognostic values in diverse malignancies. Inhibition of PARP1 by chemical inhibitors such as olaparib or rucaparib has demonstrated treatment efficacy in *BRCA1/2* mutant tumors [34]. Lai et al. [35] reported that depletion of PARP1 with siRNA diminishes PCa cell progression regardless of the *BRCA1/2* mutation. PARP1 silencing reduces cell migration and invasion in vitro and inhibits tumor growth in a PC3 xenograft model. Notably, knockdown of PARP1 increases the induction of apoptosis in PCa treated with docetaxel, implying that PARP1-siRNA may be a potential therapeutic agent against PCa by application either alone or in combination with other therapeutic tools [35]. 

Fusion genes are a class of oncogenes that have been found in many cancer types including PCa and caused by genomic rearrangements [36]. Gene fusion between a transcription repressor (*BMI1*) and a transcriptional factor (*COMMD3*) has been recently identified in PCa, which triggers disease recurrence and poor survival [37]. It was reported that targeting the *COMMD3:BMI1* fusion gene with siRNA leads to reduced c-MYC expression and decreased tumor cell proliferation both in vitro and in metastatic tumors in a xenograft mouse model of PCa [37]. Moreover, *TMPRSS2:ERG* gene fusion is the most common genomic alteration identified in PCa, leading to overexpression of the transcription factor ERG [38]. It was shown that TMPRSS2-ERG knockdown mediated by siRNA declines cell viability and inhibits tumor growth. Treatment with flutamide, one of the gold-standard treatments of PCa, and TMPRSS2-ERG siRNA results in similar inhibition of tumor growth in tumor-bearing mice, signifying high efficacy of siRNA treatment in impeding PCa progression [38]. 

Several cell membrane proteins that are overexpressed in PCa have been shown to play decisive roles in cell development and tumor progression. For example, transmembrane channel-like 5 (TMC5) is upregulated in PCa cells, and knockdown of TMC5 using siRNA leads to inhibition of PCa cell proliferation through cell cycle arrest at the G1 phase [39]. Moreover, cell sensitivity to 5-fluorouracil is remarkably enhanced after silencing TMC5 [39]. Similarly, T-type calcium channels were revealed to be a promising target for different cancers, particularly PCa, in light of their role in tumor growth. SiRNA-based inhibition of these particular calcium channels was able to lessen PC-3 cell survival and proliferation [40]. Transient receptor potential melastatin 2 is another calcium-permeable ion channel and considered as a prognostic marker for PCa [41]. Gene silence of this receptor through siRNA transfection results in altered expression of autophagic genes in PC-3 cells [41]. Nicotinic acetylcholine receptor subunit α5 (α5-nAChR) was proven to participate in the pathogenesis of certain solid tumors through induction of angiogenesis and metastasis [42]. To assess the precise role of α5-nAChR in PCa, siRNA was used to silence α5-nAChR in PCa cell lines, DU145 and PC3. Results demonstrated that knockdown of α5-nAChR causes reduced levels of phospho-AKT and phospho-ERK1/2, followed by a significant decrease in cell migration and invasion and an induction of apoptosis, indicating the impact of α5-nAChR on the proliferation and invasion of human PCa cells and the importance of its silencing in PCa treatment [43]. Finally, six transmembrane epithelial antigens of the prostate 1 (STEAP1) was shown to be overexpressed in various types of tumors, particularly in PCa, and knockdown of STEAP1 in LNCaP cells by siRNA decreases cell viability and proliferation, whilst promoting apoptosis [44]. All together, these data suggest that cell membrane proteins could serve as appropriate targets for PCa treatment.

Hypoxia is one of the key characteristics of tumors and correlates with poor prognosis of cancer patients. Dai et al. [45] demonstrated that hypoxia is capable of elevating metastatic-associated cell functions via activating c-Src in PCa cells and that gene silence of Src with siRNA impairs hypoxia-induced metastasis, implying an important role for Src in the progression of prostate malignancy. Pyruvate kinase M2 (PKM2) is a vital enzyme in aerobic glycolysis in normal tissues; however, overexpression of this protein has been reported in various cancers. Knockdown of PKM2 via siRNA was reported to reduce viability and colony formation ability of human PCa DU145 cells [46]. Further investigations revealed that depletion of PKM2 has a major impact on the PKB/mTOR pathway; hence, as a result, reducing the expression of glycolytic enzymes lactate dehydrogenase A and glucose transporter 1. In addition, an enhancement in autophagic cell death was observed after treating cancer cells with PKM2-siRNA, signifying new perspectives in terms of PCa therapy [46]. Consistent with these findings, it was demonstrated that knockdown of hypoxia-inducible factor-1 alpha (HIF-1α) by siRNA elicits antitumor activity of cisplatin in a PC-3 xenograft model [47]. Intravenous injection of attenuated Salmonella carrying an HIF-1α siRNA-expressing plasmid in tumor-bearing mice increases the response of PCa cells to cisplatin through promoting the production of reactive oxygen species (ROS) [47].

There are several proteins in cells that are overexpressed during prostate malignancy and play crucial roles in both tumor formation and invasion. For instance, the association between overexpression of a small ribosomal protein subunit 7 (RPS7) with tumor growth has been highlighted in various studies [48,49]. It was reported that silencing RPS7 using specific siRNA attenuates prostate tumor growth and migration, associated with upregulation in E-cadherin and downregulation in N-cadherin and Snail [50]. Sal-like 4 is a transcription factor that is upregulated in several types of cancers [51]. Research revealed that targeting Sal-like 4 with specific siRNA decreases proliferation and colony formation, and induces apoptosis in PCa C4-2 cells, likely through regulation of the expression of Bcl-2 and Bax [52]. Macrophage-capping protein (CAPG), encoded by the CapG gene, is recognized as an actin regulatory protein [53]. Li et al. [54] showed that silence of CapG using siRNA significantly reduces migratory and invasive capacities of DU145 cells and leads to a noticeable reduction in proliferation and metastasis of DU145 cells. ELK3 is an ETS domain-containing transcription factor involved in diverse physiological and pathological processes from cell proliferation and migration to malignant progression [55]. Silencing ELK3 by siRNA results in increased apoptosis and decreased cell proliferation and migration in DU145 cells, partially owing to upregulation of an endogenous serine protease inhibitor, as well as causes reduced tumor growth in xenograft mice [56]. The histone chaperone protein, anti-silencing function 1B (ASF1B), is also highly expressed in PCa tissues and siRNA knockdown of ASF1B was shown to result in a significant decline in viability and colony formation as well as an increase in apoptosis and cell cycle arrest of both LNCap and C4-2 cells [57].

Another imperative factor in tumor progression is a proteoglycan protein named endothelial cell-specific molecule-1 (ESM-1). It is overexpressed in various malignancies and mediated by inflammatory cytokines and proangiogenic growth factors [58]. Interestingly, it was verified that treatment of PCa cells with ESM-1-siRNA significantly diminishes cell migration with no observable impacts on proliferation [59]. Further investigations revealed that ESM-1-siRNA downregulates the transcriptional and protein levels of the angiogenic chemokine CXCL3, suggesting a role for CXCL3 in ESM-1-mediated cell migration [59].

In summary, there are myriad genes that are overexpressed during prostate malignancy and participate actively in cell proliferation, invasiveness, and tumor progression. Targeting these upregulated genes offers a promising strategy for preventing tumor progression and increasing survival among patients suffering from PCa. As validated in various studies, designing and developing siRNAs that can effectively silence genes involved in tumor pathogenesis has shown encouraging outcomes in PCa treatment. Hence, they might be applied as alternative tools to conventional treatments or be used in combination with other treatments to improve their efficacy. Table 1 summarizes different genes that are involved in PCa formation and pathogenesis, which could be potential targets for siRNAs.

## 4. Attenuating Drug Resistance in Prostate Cancer Using siRNA

Drug resistance in tumor cells remains the central cause of treatment failure, leading to tumor recurrence and metastasis [60]. Drug resistance in cancer occurs due to one of these two scenarios: inherent resistance (without any drug treatment due to genetic mutations) or acquired drug resistance (as a result of tumor adaptation following cancer treatment) [60]. Combination therapies particularly targeting genes involved in drug resistance have emerged as novel strategies for cancer therapy [61]. Castration-resistant prostate cancer (CRPC) is associated with the vast majority of PCa-related deaths worldwide. Patients suffering from CRPC have typically shown resistance to common chemotherapeutic agents. Recently, combination therapies using chemotherapeutic drugs and siRNA have served as a promising procedure for the treatment of drug-resistant PCa. In line with this, Zhang et al. [62] developed a nanoparticle delivery system, consisting of a calcium phosphate core, dioleoyl phosphatidic acid and arginine-glycine-aspartic acid peptide-modified polyethylene glycol, for co-delivery of the 78-kDa glucose-regulated protein (GRP78)-specific siRNA and docetaxel as a combination therapy against PC-3 CRPC. Results signify that co-administration of docetaxel and GRP78-siRNA to PC-3 cells elicits enhanced sensitivity of cancer cells to docetaxel both in vitro and in vivo, which might be a result of cell cycle arrest, apoptosis, and autophagy mediated by GRP78 knockdown [62]. Similar to this, knockdown of deubiquitinating enzyme ubiquitin-specific protease 33 (USP33), which is overexpressed in PCa cells, with siRNA considerably induces apoptosis mediated by docetaxel in CRPC [63]. In addition, siRNA-mediated knockdown of USP9X notably decreases anchorage-independent growth of prostate carcinoma cells, possibly through increasing ubiquitination and decreasing protein levels of IGFR (insulin-like growth factor receptor) and IRS-2 (insulin receptor substrate-2), indicating the importance of targeting certain ubiquitin-specific proteases in PCa treatment [64]. The gene associated with retinoid-interferon mortality (GRIM-19) has been shown to be related to drug resistance in a number of cancers [65]. The mRNA and protein levels of GRIM-19 were reported to be lower in PCa tissues and cells compared with those in normal tissues [65]. Downregulation of GRIM-19 with designed siRNA increases the resistance of PCa cells to docetaxel, whilst overexpression of GRIM-19 improves the sensitivity through downregulating the expression of Rad23b, a survival gene that facilities DNA damage repair [65]. These data suggest that enhanced expression of GRIM-19, in combination with chemotherapy, could serve as a better strategy of chemotherapy for PCa. Epidermal growth factor receptor (EGFR) is a well-known factor in prostatic tumorigenesis; however, its role in chemoresistance in human PCa is still ambiguous. Hour et al. [66] revealed that there is a direct association between EGFR level and docetaxel resistance in docetaxel-resistant PCa cells, which could occur through Akt-dependent ABCB1 expression in PC cells. It was proven that silencing EGFR expression through siRNA can significantly increase docetaxel sensitivity of docetaxel-resistant PCa cells, while induction of EGFR expression or applying recombinant EGF protein diminishes cytotoxicity of docetaxel toward PC3 cells [66]. Together, these studies have provided increasing evidence that combined administration of chemotherapeutic agents with specific siRNAs can augment the function of chemotherapeutic drugs and recover the sensitivity of resistant cells to conventional drugs such as docetaxel.

## 5. Improving Antitumoral Immune Response by siRNA

In recent years, cancer immunotherapy has gained vast attention in light of its capabilities of augmenting the host immune response towards cancer cells [67]. siRNA has emerged as a powerful approach for cancer immunotherapy [68]. siRNA can target different molecules, such as programmed cell death-1 (PD-1) and its ligand (PD-L1), as well as interleukin 10 receptor, STAT3, and transforming growth factor-β receptor, on the surface of immune cells and/or tumor cells, which participate actively in the process of immune activation, to lessen tumor evasion from the host’s immune system and boost the immune response against tumors [69,70,71,72]. Consistently, myeloid-derived suppressor cells (MDSC) are recognized as imperative inhibitors of T-cell responses in numerous malignancies, including PCa. Therefore, alleviation of MDSC-mediated immunosuppression is an effective method to restore immune activity against cancer. It has been confirmed that STAT3 is activated in circulating MDSCs and has a decisive role in MDSC-mediated immunosuppression [73]. It was shown that immunosuppressive impacts of patient-derived MDSCs on effector CD8 (+) T cells can be abrogated through delivery of STAT3-siRNA to MDSCs [74]. Further investigation revealed that inhibitory effects of STAT3-siRNA are contingent on decreased expression and enzymatic activity of arginase-1, a downstream target gene of STAT3 and a potent T cell inhibitor [74]. These results indicate that gene silencing of STAT3, a central immune checkpoint regulator, is a promising strategy to enhance the immune response against PCa. Moreover, the E3 ubiquitin ligase Cbl-b is expressed in all leukocyte subsets and mediates various signaling pathways. Targeting the Cbl-b gene in T lymphocytes by specific siRNA was reported to increase the production of interleukin (IL)-2 and interferon (IFN)-γ and stimulate T cell cytotoxicity towards RM-1 PCa cells, resulting in a noticeable decline of tumor size in immune competent mice compared with controls [75]. Collectively, despite these promising results, further investigations are required to identify more molecules that are associated with tumor evasion to design siRNA in order to target them with high selectivity.

## 6. Targeted siRNA Delivery

**Figure 1:**
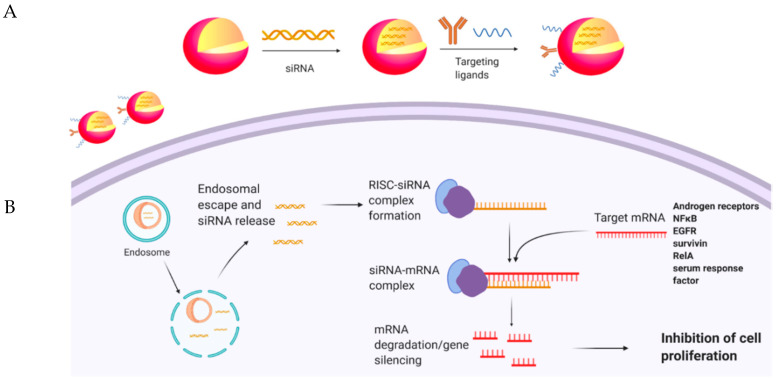
(**A**) Encapsulation of siRNAs in nanoparticles, modified with targeting ligands. (**B**) Delivery and release of siRNAs to desired cells, leading to gene silencing.

Despite the fact that siRNA therapy is a promising strategy for cancer treatment, there are some concerns regarding their applications in clinical therapy. Naked siRNAs have very short half-lives in vivo and undergo rapid clearance [76]. Moreover, they induce immune responses after in vivo injection [77]. In recent years, advancements in the field of nanotechnology and progress in designing nanocarriers have significantly improved the efficacy of siRNA delivery into cancer cells [78] (Figure 1). For instance, Shi et al. [79] developed a nanocarrier system based on human monoclonal prostate-specific membrane antigen antibody (PSMAab) for targeted delivery of tripartite motif-containing 24 (TRIM24)-siRNA. It was proven that developed nanoparticles not only protect siRNA from enzymatic digestion, but also efficiently deliver siRNA into PCa cells in vitro and in vivo. Release of TRIM24-siRNA, followed by knockdown of TRIM24, significantly suppresses proliferation, colony-formation, and invasion of PSMA+ CRPC cells in vitro, and inhibits tumor growth of PSMA+ CRPC xenografts and bone loss in a PSMA+ CRPC bone metastasis model [79]. Similarly, Lee et al. [80] developed a delivery system composed of Glu-urea-Lys PSMA-targeting ligand/siRNA incorporated into a lipid nanoparticle to target androgen receptors on the surface of PCa. It was demonstrated that, compared with naked siRNA, this system is able to decrease serum prostate-specific antigen, tumor cellular proliferation, and androgen receptor levels significantly. This implies the importance of a targeted delivery system compared with nontarget strategies for treating PCa cells with siRNA [80]. 

Owing to their unique and easy modification properties, dendrimers have emerged as a promising system for gene/drug delivery in cancer therapy [81,82]. For instance, it has been shown that encapsulation of Hsp27-sticky siRNA in dendrimers significantly enhances the efficacy of gene silencing in different prostate cancer cells [83]. Research by the same group revealed that further modification of these dendrimers with the RGDK peptide allows for selective targeting of ανβ3 integrin and neuropilin-1 (Nrp-1) receptors, which are overexpressed on tumor cells, and effectively prevents tumor cell proliferation in a PC-3 xenograft mouse model [81]. Carbon nanotubes are another type of nanoparticles that are widely applied as superior carriers for siRNA due to their exceptional characteristics such as large surface areas, rich surface chemical functionalities, and good biocompatibility [84]. siRNAs can be attached on the surface of carbon nanotubes through both electrostatic and covalent interaction and effectively delivered to desired regions [85]. Alongside these nanocarriers, it was shown that targeted delivery of NFκB-siRNA through gold nanoparticles capped with polyethylenimine (PEI) and PEGylated anisamide (a ligand known to target the sigma receptor) causes effectual endosomal escape of siRNA and subsequently reduces NFκB gene expression [86]. As a result, considerable tumor growth suppression in a PC-3 xenograft mouse model was achieved. Furthermore, systemic exposure to siRNA parallel with paclitaxel delivery results in a synergistic therapeutic response in terms of tumor growth inhibition [86]. In another study, to evaluate the effect of a combination therapy, docetaxel and ERK1/2-siRNA were loaded into PSMA-conjugated bovine serum albumin-branched polyethylenimine nanoparticles [87]. Co-delivery of docetaxel and ERK1/2-siRNA leads to a marked decrease in α-tubulin and ERK1/2 in CWR22R PCa cells. As a result, cell growth is significantly inhibited as compared to docetaxel alone. Moreover, combined administration of docetaxel and ERK1/2-siRNA remarkably enhances median survival from 18 days to around 45 days in comparison with mice that receive the same dose of free docetaxel. These findings signify the impact of a combined therapy involving siRNA and conventional chemotherapy drug and the development of an effective delivery system in the treatment of PCa [87]. 

Both the EGFR signaling pathway and survivin are involved in cancer cell proliferation, tumor vascularization, and metastasis [88]. Lui et al. [89] constructed an RNA-based aptamer-siRNA chimera that specifically binds PSMA and targets both EGFR and survivin. This chimera was shown to induce apoptosis effectively both in vitro and in vivo, and inhibit tumor growth and angiogenesis in the C4-2 PCa xenograft model by an EGFR-HIF1α-VEGF-dependent mechanism. These results support that targeted delivery of two siRNAs could be a therapeutic strategy for the treatment of PCa [89]. Serum response factor (SRF) is known as a vital transcription factor and has a significant role in regulating expression of genes involved in cell growth and differentiation [90]. Research revealed that knockdown of both RelA (also known as nuclear factor NFκB p65 subunit) and SRF by specific siRNAs through a non-virally modified cyclodextrin vector causes significant reductions in the invasion potential of the highly metastatic PC-3 cells, partly due to the reduction of MMP-9 [91]. Similar results in terms of a decrease in cell viability of PC-3 cells were achieved through a delivery of siRNAs targeting disintegrin and metalloproteinase 10 (ADAM10) by polyethylene glycol–polyethylenimine–Fe3O4 nanoparticles [92]. Furthermore, Choi et al. [93] discovered that gene silence of Bcl-2, survivin, and androgen receptor with siRNAs, which are encapsulated in pluronic F-68 polymer, along with treatment with benzethonium chloride, triggers apoptosis of human PCa LNCaP-LN3 cells more efficiently in comparison with the silencing of individual gene, suggesting that multiple gene-targeting siRNA/drug delivery system could be a feasible and promising way to combat PCa [93]. Taken together, results obtained by these studies and other investigations in different malignancies revealed that a nanoparticle-mediated delivery system may serve as an effective platform for combined gene therapies with conventional chemotherapies to enhance antitumoral efficacy. Table 2 summarizes recent delivery platforms that are employed for effective and safe delivery of various siRNAs to PCa cells.

## 7. Conclusions

Since its discovery, siRNA holds great promise to treat a number of diseases, including cancer, owing to its specificity to target and silence genes that are correlated with the pathogenesis of cancer, from cell proliferation and invasion to immunosuppression [94]. Recently, combinatory administration of gene-specific siRNAs with other conventional therapies such as chemotherapy and radiotherapy has shown synergistic effects in the treatment of PCa [79]. More importantly, several studies confirmed that targeting specific genes by siRNA could noticeably restore or even enhance the sensitivity of resistant PCa cells to chemotherapeutic drugs such as docetaxel [95]. Clinical studies have been initiated to evaluate the efficiency of siRNA in various solid tumors either alone or in combination with other therapeutic agents (NCT03087591, NCT00672542). Here we summarized the molecules that could be potential targets of siRNAs for PCa treatment. Despite the promising results, the clinical application of naked siRNAs has demonstrated some limitations because of their labile nature, ability to induce immune responses, and anionic properties [96]. To protect siRNAs from enzymatic cleavage and rapid clearance, it is imperative to shield them in a proper way. Advancements in the field of nanotechnology have provided this opportunity for efficient delivery of siRNAs to target cells and to increase their in vivo cellular uptake [97,98]. Moreover, it is beneficial in terms of combining siRNAs with other therapeutic agents [99]. Further investigations in molecular profiling of patient tumors are needed to determine molecules that are involved in tumor pathogenesis and could be potentially targeted by siRNA, and to explore novel strategies for delivery of siRNAs with high selectivity and safety.

## Figures and Tables

**Table 1:** Potential targets for siRNA treatment in prostate cancer.

SiRNA-Target Gene	Knockdown Consequences	Ref.
Dual specificity protein kinase TTK	Reduces proliferation, invasion, and migration, as well as initiates cell death process in PC-3 and DU145 PCa cells.	[31]
BMI1: *COMMD3* fusion gene	Diminishes c-MYC expression in PC-3 cells resistant to BRD/BET-inhibitor and suppresses metastasis of tumor in xenograft mouse models.	[37]
TMPRSS2:ERG fusion gene	Declines cell viability and inhibits tumor growth of VCaP PCa cells.	[38]
Transmembrane channel-like 5 (TMC5)	Inhibits cell proliferation and enhances cell sensitivity to 5-fluorouracil in PC-3 and DU145 cells.	[39]
T-type calcium channels	Lessens cell survival and proliferation of PC-3 cells.	[40]
Transient receptor potential melastatin 2 (TRPM2)	Induces autophagy in PC-3 cells.	[41]
Src	Impairs hypoxia-induced metastasis of PC-3ML and C4-2B cells.	[45]
Pyruvate kinase M2 (PKM2)	Inhibits cell viability and the ability of colony formation, as well as induces autophagic cell death in DU145 cells.	[46]
Rho-associated protein kinase (ROCK)	Reduces migration and invasion of PC-3 and DU145 cells.	[32]
Protein phosphatase 2A (PP2A)	Elicits sensitivity of PC-3 cells to docetaxel.	[33]
Poly (ADP-ribose) polymerase 1 (PARP-1)	Reduces PC-3 cell migration and invasion, and decreases xenograft tumor size.	[35]
Endothelial cell-specific molecule-1 (ESM-1)	Diminishes cell migration with no impact on proliferation of PC-3 cells.	[59]
Small ribosomal protein subunit 7	Attenuates PCa growth and migration of PC-3 cells.	[50]
Sal-like 4 (SALL4)	Decreases proliferation and colony formation capacity of C4-2 cells.	[52]
Macrophage-capping protein (CAPG)	Reduces proliferatory, migratory, and invasive capacities of DU145 cells	[54]
Nicotinic acetylcholine receptor (nAChR)	Decreases cell migratory and invasive activities, and induces apoptosis of DU145 and PC-3 cells.	[43]
Six transmembrane epithelial antigen of the prostate 1 (STEAP1)	Declines cell viability and proliferation whilst promoting apoptosis of LnCap PCa cells.	[44]

**Table 2:** siRNA delivery strategies.

siRNA Target	Delivery Platform	Effects	Ref.
Tripartite motif-containing 24	PSMAab	Suppresses proliferation, colony formation, and invasion of PSMA+ CRPC cells in vitro, and inhibits tumor growth of PSMA+ CRPC xenografts and bone loss in a PSMA+ CRPC bone metastasis model.	[79]
Androgen receptor	Glu-urea-Lys PSMA-lipid nanoparticle	Inhibits serum prostate-specific antigen, tumor cellular proliferation, and androgen receptor levels.	[80]
NFκB	Gold nanoparticle-PEI PEGylated anisamide	Suppresses tumor growth in a PC-3 xenograft mouse model. Its combination with paclitaxel leads to a synergistic therapeutic response in terms of tumor growth inhibition.	[86]
p44/42 mitogen-activated protein kinase	PSMAab-Bovine Serum Albumin branched polyethylenimine	Inhibits cancer cell proliferation.	[87]
EGFR and survivin	RNA-based aptamer-siRNA chimera	Induces apoptosis both in vitro and in vivo, and diminishes tumor growth and angiogenesis in the C4-2 PCa xenograft model.	[89]
RelA and serum response factor	Non-viral modified cyclodextrin vector	Reduces metastatic potential of PC-3 cells without noticeable impacts on cell viability.	[91]
